# ATP-dependent chromatin remodeling in the DNA-damage response

**DOI:** 10.1186/1756-8935-5-4

**Published:** 2012-01-30

**Authors:** Hannes Lans, Jurgen A Marteijn, Wim Vermeulen

**Affiliations:** 1 Department of Genetics, Medical Genetics Center, Erasmus MC, PO Box 2040, 3000 CA Rotterdam, The Netherlands

## Abstract

The integrity of DNA is continuously challenged by metabolism-derived and environmental genotoxic agents that cause a variety of DNA lesions, including base alterations and breaks. DNA damage interferes with vital processes such as transcription and replication, and if not repaired properly, can ultimately lead to premature aging and cancer. Multiple DNA pathways signaling for DNA repair and DNA damage collectively safeguard the integrity of DNA. Chromatin plays a pivotal role in regulating DNA-associated processes, and is itself subject to regulation by the DNA-damage response. Chromatin influences access to DNA, and often serves as a docking or signaling site for repair and signaling proteins. Its structure can be adapted by post-translational histone modifications and nucleosome remodeling, catalyzed by the activity of ATP-dependent chromatin-remodeling complexes. In recent years, accumulating evidence has suggested that ATP-dependent chromatin-remodeling complexes play important, although poorly characterized, roles in facilitating the effectiveness of the DNA-damage response. In this review, we summarize the current knowledge on the involvement of ATP-dependent chromatin remodeling in three major DNA repair pathways: nucleotide excision repair, homologous recombination, and non-homologous end-joining. This shows that a surprisingly large number of different remodeling complexes display pleiotropic functions during different stages of the DNA-damage response. Moreover, several complexes seem to have multiple functions, and are implicated in various mechanistically distinct repair pathways.

## DNA-damage response

All living organisms depend on faithful preservation and transmission of genetic information to the next generation. Genetic information is stored within DNA, which is embedded in a dynamic nucleoprotein complex, called chromatin. The integrity of DNA is inescapably and continuously threatened by spontaneous and induced alterations to its basic structure. DNA itself is unstable and undergoes hydrolysis, which creates abasic sites and causes deamination [1]. Furthermore, cellular metabolic processes such as oxidative respiration produce oxygen radicals and other reactive molecules, which damage DNA [2]. Finally, exposure to environmental sources such as solar UV irradiation, × radiation, and numerous chemicals induces DNA injuries.

DNA damage interferes with vital processes such as transcription and replication, which may cause cells to die or senesce, thus contributing to aging [3]. Replication of damaged DNA templates severely affects the fidelity of the polymerases, and may result in permanent mutations or chromosomal aberrations, which are at the basis of malignant transformation. Genetic erosion and its consequences are neutralized by a variety of DNA repair and associated DNA-damage signaling pathways, collectively called the DNA-damage response (DDR) [3-6]. In this review, we will focus on three repair pathways which are among the best characterized with regard to their repair mechanisms and interactions with chromatin: nucleotide excision repair (NER), which removes helix-distorting intra-strand lesions, and homologous recombination (HR) and non-homologous end-joining (NHEJ), both of which repair double-strand breaks (DSBs).

## Chromatin and the DNA-damage response

All DNA-associated processes, such as transcription, replication, recombination, and DNA repair, are for a large part regulated by the chromatin structure [7,8]. Because this nucleoprotein complex limits the ability of other proteins to interact with DNA, the chromatin structure needs to be modified to facilitate efficient access to DNA. In addition, DNA processing itself may also lead to changes in the chromatin structure and composition, such as DNA repair-associated post-translational histone modification and exchange. Based on the changes in chromatin observed after NER, it was suggested that chromatin needs to be made more accessible in order to allow efficient damage detection and repair to take place, after which the chromatin structure is restored to prevent genomic instability and possibly to preserve epigenetic information or to leave an imprint of the damage on the chromatin. Particularly for the latter step, clear evidence is available that nucleosome occupancy of repaired DNA is restored by the action of histone chaperones [9-12]. However, evidence for chromatin remodeling before or during repair seems to be more ambiguous, and difficult to include in a single overarching model.

Modification of chromatin basically occurs via two mechanisms. First, chromatin can be changed by post-translational modification of histones through the action of histone-modifying enzymes. Second, chromatin can be altered by displacement of histones or entire nucleosomes through the action of ATP-dependent chromatin-remodeling complexes and histone chaperones. Although both mechanisms are interconnected, in this review we will mainly discuss the second mechanism and focus on mammalian cells, although current knowledge in yeast will also be described. Readers interested in DDR-associated post-translational histone modifications are referred to other recent reviews on the subject [6,13-15].

## ATP-dependent chromatin-remodeling complexes

A large array of different chromatin-remodeling complexes has been identified, and these play important roles in controlling gene expression by regulating recruitment and access of transcription factors [16]. In recent years, it has become clear that ATP-dependent chromatin-remodeling complexes also play important roles in the DDR. Using the energy of ATP hydrolysis, these complexes catalyze disruption of DNA-histone contacts, and can slide or evict nucleosomes or alter their composition [17-19]. Among the different complexes identified in different species, four structurally related families have been described: SWI/SNF (switching defective/sucrose nonfermenting), INO80 (inositol requiring 80), CHD (chromodomain, helicase, DNA binding) and ISWI (imitation switch) (Table 1). Each family is defined by its characteristic catalytic core ATPase enzyme from the SWI2/SNF2 superfamily. ATP-dependent chromatin-remodeling complexes have diverse but specific functions in processes involving DNA metabolism. Although some redundancy occurs, most remodelers are essential for cellular growth, development, or differentiation. Consequently, they have been implicated in many embryonic developmental processes and in tumorigenesis [17,18].

**Table 1:** Yeast and mammalian ATP-dependent chromatin remodeling proteins and complexes implicated in the DDR.

Family	Complex	ATPase	Additional subunits	Species	Ref
SWI/SNF	SWI/SNF	Snf2	Swi3, Snf12, Arp7, Arp9, Snf11, Snf5, Snf6, Swi1, Swp82, Taf14	Yeast	[157]
	RSC	Sth1	Rsc1-4, Rsc6, Rsc8-, Rsc9, Rsc30, Rsc58, Rsc7, Arp7, Arp9, Sfh1, Htl1, Ldb7, Rtt102	Yeast	[157]
	BAF	SMARCA4/BRG1, SMARCA2/BRM	ARID1A, SMARCC2, SMARCC1, SMARCD1-3, SMARCE1, ACTL6A, SMARCB1/SNF5, BAF45a-d, BRD7, BRD9, ACTB	Mammal	[17,157]
	PBAF	SMARCA4/BRG1, SMARCA2/BRM	ARID2, PBRM1, SMARCC2, SMARCC1, SMARCD1-3, SMARCE1, ACTL6A, SMARCB1/SNF5, BAF45, BRD7, BRD9, ACTB	Mammal	[157]
INO80	INO80	Ino80	Arp4, Arp5, Arp8, Act1, Rvb1, Rvb2, Taf14, Ies1-6, Nhp10	Yeast	[158]
	SWR1	Swr1	Arp4, Arp6, Act1, Rvb1, Rvb2, Yaf9, Vps71, Vps72, Swc3-5, Swc7, Bdf1	Yeast	[17,158]
	INO80	INO80	RUVBL1, RUVBL2, ACTL6A, ACTR5/Arp5, ACTR8/Arp8, INO80B, INO80C, TFPT, NFRKB, MCRS1, INO80E, INO80D	Mammal	[17]
	TRRAP/Tip60^1^	EP400/p400	RUVBL1, RUVBL2, KAT5/Tip60, ACTL6A, ACTB, YEATS4, DMAP1, VPS72, BRD8, MORF4L1, MORF4L2, MEAF6, C20orf20, EPC1, EPC2, ING3	Mammal	[17]
CHD	NuRD	CHD3, CHD4	MBD2, MBD3, MTA1-3, HDAC1, HDAC2, RBBP7, RBBP4, GATAD2A, GATAD2B	Mammal	[17,159]
		CHD2^2^		Mammal	[137]
ISWI	ISWIa	Isw1	Ioc3	Yeast	[160]
	ISWIb	Isw1	Ioc2, Ioc4	Yeast	[160]
	ISW2	Isw2	Itc1, Dpb4, Dls1	Yeast	[17,160]
	ACF	SMARCA5/hSNF2H	BAZ1A/hACF1	Mammal	[17,160]
	CHRAC	SMARCA5/hSNF2H	BAZ1A/hACF1, CHRAC1, POLE3	Mammal	[17,160]
	WICH	SMARCA5/hSNF2H	BAZ1B/WSTF	Human	[17,160]
	NURF	SMARCA1/hSNF2L	BPTF, RBBP7, RBBP4	Mammal	[17,160]
Uncategorized		Rad16		Yeast	[48,49]
		Rad26		Yeast	[161]
		Rad5		Yeast	[162]
		Rdh54		Yeast	[163,164]
		CHD1L/ALC1		Mammal	[132,133]
		ERCC6/CSB		Mammal	[165]
		HLTF		Mammal	[166,167]
		RAD54L		Mammal	[90,168]
		RAD54B		Mammal	[169-171]
		SHPRH		Mammal	[167,172]
		SMARCAL1		Mammal	[173,174]

See main text for references in which the different ATP-dependent chromatin-remodeling complexes are implicated in the DNA-damage response.

## Chromatin and nucleotide excision repair

**Figure 1:**
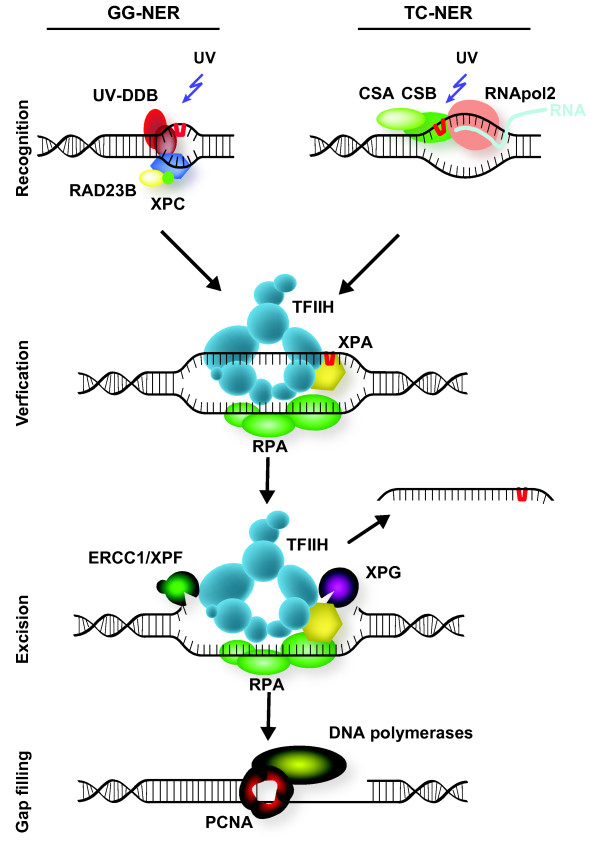
**Mammalian nucleotide excision repair (NER)**. NER removes a wide variety of helix-distorting lesions from DNA, including those induced by UV light [20]. NER is executed by two different damage-detection mechanisms, which utilize the same machinery to excise and repair the damage. Transcription-coupled NER (TC-NER) is initiated by stalling of RNA polymerase 2 on a lesion present in the transcribed strand of active genes, and depends on recruitment of the CSA and CSB proteins. Other lesions are removed by global genome NER (GG-NER), which is initiated by the UV-DDB ubiquitin ligase complex and the heterotrimeric XPC/RAD23/CETN2 complex. Following detection, the transcription factor II H (TFIIH) complex is recruited, and unwinds a stretch of approximately 30 nucleotides around the damage, providing access for other repair factors. The DNA-binding proteins XPA and RPA are thought to stimulate the translocation and damage-verification activity of TFIIH and to stabilize and orient the XPF/ERCC1 and XPG endonucleases, which subsequently incise the DNA around the damage. After excision of the damaged strand, the resulting gap of 25 to 29 nucleotides is filled in by DNA synthesis and ligation, involving replication factors such as PCNA and RFC, DNA polymerases δ, ε and κ, and final sealing of the gap by DNA ligases I and III. For clarity, not all proteins known to be involved in NER are shown.

NER removes a wide range of bulky DNA adducts that distort the double helix, including those induced by UV light. NER is initiated by two damage-recognition pathways, transcription-coupled (TC)-NER and global genome (GG)-NER, which remove damage in roughly four successive steps: 1) lesion detection, 2) local unwinding and damage verification, 3) excision of the DNA surrounding the lesion, and finally 4) DNA synthesis and ligation to fill the resulting gap (Figure 1) [20]. Damage that occurs in the transcribed strand of active genes is mainly repaired by TC-NER, whereas damage in non-transcribed DNA is repaired by GG-NER.

The importance of chromatin remodeling for NER is apparent from many experiments that have shown that NER is more efficient in naked DNA than in chromatin, and is inhibited by the presence of nucleosomes and heterochromatin, which limit the access of repair proteins to DNA [11,21-23]. Thus, for NER to recognize, excise, and repair DNA damage efficiently, the chromatin needs to be adapted. Conversely, DNA injuries may differentially affect chromatin structure. Although NER removes a large variety of structurally unrelated DNA lesions, including UV-induced cyclobutane pyrimidine dimers (CPDs) and 6-4 photoproducts (64PPs) [24], removal efficiency of these lesions differs significantly. For example, in non-transcribed DNA, GG-NER of 64PPs is much faster than GG-NER of CPDs [25-27]. Chromatin may exert substantial influence on this lesion-specific repair, as 64PPs seem to be predominantly formed in internucleosomal regions, whereas CPDs are formed equally in nucleosomal and internucleosomal DNA [28]. Nevertheless, in naked DNA [29] and nucleosomes [30], 64PPs are also repaired faster than CPDs. Lesion-specific differences in NER efficiency have also been explained by the degree to which they distort the helical DNA structure and define the affinity for the GG-NER initiation factor XPC [31,32]. Furthermore, repair of CPDs in non-transcribed DNA depends on the presence of the GG-NER-specific DNA damage-binding (DDB) recognition complex, which is thought to modify chromatin to facilitate binding of XPC [33-38]. In TC-NER, which is initiated upon stalling of RNA polymerase 2 after damage, different lesions are removed with equal speed [26,39]. This suggests that differences in repair rate are caused not only by the helix-distorting capacity and chromatin environment, but also by the manner in which lesions are detected. Lesion removal in transcribed DNA occurs more rapidly than in non-transcribed DNA [40], possibly because both GG-NER and TC-NER remove these lesions, but possibly also because chromatin has already been made more accessible by the transcription machinery.

## ATP-dependent chromatin remodeling during nucleotide excision repair

UV damage itself enhances unwrapping of nucleosomes, which normally exist in a dynamic equilibrium between wrapping and unwrapping [41]. This enhanced 'DNA breathing' may assist the repair of lesions in chromatin by increasing the time window for repair factor access and binding of these repair proteins to lesions might further unwrap the DNA. Additionally, access to DNA is likely to be actively induced by multiple ATP-dependent chromatin-remodeling complexes, which have been implicated in the response of cells to UV irradiation (Table 1). One of the major TC-NER proteins, CSB/ERCC6, contains a SWI2/SNF2 ATPase domain, which is essential for recruitment of the protein to chromatin and to its function in repair [42-45]. CSB is able to remodel chromatin *in vitro *in an ATP-dependent fashion [46], and is required for the recruitment of NER factors to sites of TC-NER [47], but its precise activity and whether it remodels nucleosomes during TC-NER *in vivo *remains unknown. Another NER factor, Rad16, which is essential for GG-NER in yeast, also contains a SWI2/SNF2 domain [48,49]. Although Rad16 does not slide or evict nucleosomes, it affects chromatin structure through its DNA translocase activity [50,51] and by controlling UV-induced histone H3 acetylation induced by the histone acetyltransferase Gcn5 [52-54].

### SWI/SNF

Accumulating evidence suggests that chromatin is remodeled by SWI/SNF complexes during NER, leading to chromatin relaxation and increased access to DNA of NER initiation factors. *In vitro*, NER-mediated excision of acetylaminofluorene-guanoside or 64PP lesions within nucleosomal templates is stimulated by purified yeast SWI/SNF [55,56]. In yeast, the NER initiation complex Rad4-Rad23, orthologous to mammalian XPC/RAD23B, interacts with Snf5 and Snf6, two SWI/SNF complex subunits, in a UV-specific manner [57]. Furthermore, Snf6 and the ATPase Swi2 are required for UV-induced increase in chromatin accessibility, which is also associated with hyperacetylation of histones H3 and H4 [52,57]. Likewise, in mammals, the SWI/SNF ATPase BRG1/SMARCA4 stimulates efficient repair of CPDs but not of 64PPs [58,59], and associates with the recognition factors DDB2 [58] and XPC [59]. However, conflicting results have been reported about whether BRG1 stimulates recruitment of XPC to sites of DNA damage or, *vice versa*, whether XPC stimulates BRG1 recruitment, which in turn stimulates recruitment of later NER factors. Finally, BRG1 may also transcriptionally regulate the UV-induced G1/S checkpoint, as loss of BRG1 leads to increased UV-induced apoptosis [60].

**Figure 2:**
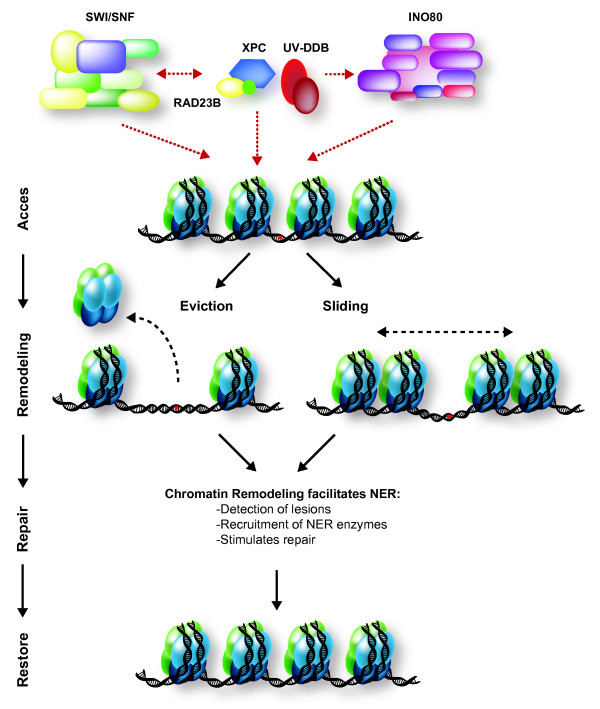
**Mammalian nucleotide excision repair (NER)-associated chromatin remodeling**. Both the SWI/SNF and the INO80 ATP-dependent chromatin-remodeling complexes are recruited to sites of UV-induced DNA damage, and are implicated in mammalian global genome NER (GG-NER). SWI/SNF may interact with the damage-detection complexes XPC/RAD23 and UV-DDB, and stimulate recruitment of XPC to the damage. Recruitment of SWI/SNF is also stimulated by XPC. In addition, mammalian INO80 interacts with UV-DDB, and stimulates recruitment of XPC. Together, SWI/SNF and INO80 are thought to regulate accessibility of DNA by sliding or eviction of nucleosomes at the damaged site. Red dotted arrows depict chromatin recruitment and protein-protein interactions. See main text for details and references.

Besides BRG1, the mammalian SWI/SNF subunit SNF5/SMARCB1 also interacts with XPC [61]. Inactivation of SNF5 causes UV hypersensitivity and inefficient CPD repair [60,62], although this has been challenged by a study in which no effect of SNF5 depletion on UV sensitivity was found [63]. These differences may be due to the use of different assays and cells. In a genetic screen, our laboratory identified different subunits of SWI/SNF complexes, including orthologs of mammalian BRG1 and BRM/SMARCA2, SNF5, PBRM1 and BAF155/SMARCC1, which are essential for optimal UV survival of somatic cells in *Caenorhabditis elegans *[64]. Intriguingly, BRG1/BRM, but none of the other subunits, is also important to the UV response of germ cells, suggesting that the involvement of individual SWI/SNF subunits may differ between cell types. Importantly, UV hypersensitivity resulting from BRG1 inactivation depends on the presence of the checkpoint protein TP53 [58], extending the complexity of the involvement of BRG1 in UV-induced DDR. How exactly SWI/SNF remodelers impinge on NER is as yet unknown, but the available data support a regulatory role in the damage-detection step of NER (Figure 2).

### INO80

The INO80 chromatin-remodeling complex probably also remodels chromatin during NER (Figure 2). Both yeast *ino80 *mutants and mammalian INO80-deficient cells are sensitive to UV irradiation [65,66]. Yeast Ino80 interacts with Rad4 and Rad23, and is recruited to damaged chromatin in a Rad4-dependent manner, where it is required for restoration of repair-induced nucleosome loss [67]. Whether yeast Ino80 is also involved in earlier repair events is controversial [67,68]. The mammalian INO80 complex functions during earlier NER steps because it facilitates the recruitment of early NER factors such as XPC and XPA, and, in contrast to yeast, localizes to damaged DNA independently of XPC [69]. Furthermore, INO80 facilitates efficient 64PP and CPD removal and, together with the Arp5/ACTR5 subunit, interacts with the NER initiation factor DDB1, but not with XPC. These discrepancies may reflect interspecies differences, but may also point to multiple functions of INO80 chromatin remodeling during NER that are experimentally difficult to dissect. INO80 may function to facilitate damage detection as well as to restore chromatin after damage is repaired.

### ISWI

Several experiments have suggested that ISWI-mediated chromatin remodeling also functions to regulate NER, although its precise role is still unknown. The recombinant *Drosophila *ISWI, ACF, stimulates NER-mediated excision of 64PP lesions in the linker DNA of reconstituted dinucleosomes, possibly through a nucleosome sliding mechanism [70]. Furthermore, we found that loss of the *C. elegans *ISWI ATPase *isw-1 *renders the nematodes sensitive to UV irradiation [64]. Knockdown of the mammalian ISWI ATPase SNF2H/SMARCA5 or its auxiliary factor ACF1/BAZ1A also leads to mild UV sensitivity [71,72]. Moreover, SNF2H interacts with CSB [73], and the ACF1 subunit is recruited to UV-induced DNA damage [74]. Further experimentation is required to understand how ISWI chromatin remodeling functions in the UV-DDR.

### Discussion and perspectives

From the above overview, it is clear that some ATP-dependent chromatin-remodeling complexes facilitate the early damage-recognition step of NER [58,69] (Figure 2), although their precise function and their interdependence is not yet clear. This early chromatin remodeling is in line with the notion that UV-induced increase of DNA accessibility, associated with histone H3 and H4 hyperacetylation, precedes NER [52,75]. Other results, however, suggest that ATP-dependent chromatin remodeling depends on active NER [59,67,76]. Interestingly, it was shown *in vitro *that not only does SWI/SNF chromatin remodeling stimulate NER, but that NER factors also stimulate chromatin remodeling activity by SWI/SNF [55]. Thus, NER initiation factors and chromatin remodelers may cooperate to stimulate each other's recruitment and activity. The earliest known recognition complex in NER, UV-DDB, was suggested to recruit chromatin-remodeling factors to sites of damage [58,69]. However, alternative UV-DDB independent mechanisms may also be involved.

Restoration on DNA of nucleosome occupancy, the loss of which results from DNA damage and its repair, may also require ATP-dependent chromatin remodeling. However, evidence for this function is still limited as to date this has only been observed for INO80 during yeast NER [67]. The yeast and mammalian histone chaperone complex CAF-1 and possibly also ASF1 are involved in this post-repair histone deposition on newly synthesized DNA as well [12,67,77-79]. Further mechanistic studies are required to address whether and how ATP-dependent chromatin remodelers regulate nucleosome occupancy after NER, and to identify the interplay with histone chaperones in this process. Furthermore, because GG-NER and TC-NER utilize alternative DNA damage-recognition mechanisms, it will be interesting to determine differences in chromatin remodeling between these NER sub-pathways, particularly because TC-NER is associated with active transcription, and chromatin is already modified to allow access to proteins. Besides involvement of SWI/SNF and INO80 remodeling complexes, the role of other complexes, such as ISWI and CHD, needs more attention. Based on the analogy to DSB repair (see below), it is expected these complexes are involved in NER as well.

## Chromatin and double-strand break repair

**Figure 3:**
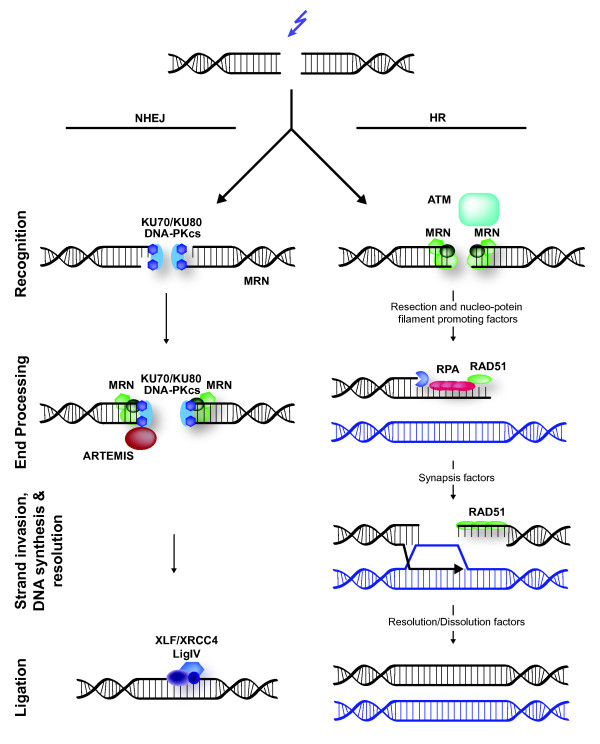
**Mammalian double-strand break (DSB) repair**. DNA DSBs are predominantly repaired by either non-homologous end-joining (NHEJ) or homologous recombination (HR) [156]. NHEJ rejoins broken DNA ends, and often requires trimming of DNA before ligation can occur. This can lead to loss of genetic information. In NHEJ, the broken DNA ends are bound by the KU70/KU80 heterodimer, which orchestrates the activity of other repair factors and recruits the phosphatidylinositol 3-kinase DNA-PKcs/PRKDC. DNA-PKcs phosphorylates and activates additional repair proteins, including itself and the ARTEMIS/DCLRE1C nuclease. ARTEMIS and/or the heterotrimeric MRE11-RAD50-NBN complex are thought to process the DNA ends prior to ligation. The DNA ends are joined by the activity of polymerases and a ligase complex consisting of XRCC4, XLF/NHEJ1 and LIG4. In contrast to NHEJ, HR is an error-free repair pathway that utilizes a sister chromatid, present only in the S- or G2-cell cycle phase, as template to repair DSBs. HR is initiated by DNA end-resection, involving the MRE11-RAD50-NBN complex and several accessory factors including nucleases. The MRE11-RAD50-NBN complex also recruits the phosphatidylinositol 3-kinase ATM, which phosphorylates histone H2AX and many other proteins involved in repair and checkpoint signaling. Single-stranded DNA generated by DNA end-resection is bound by RPA, which is subsequently replaced by RAD51. RAD51 promotes the invasion of the single-stranded DNA to a homologous double-stranded DNA template, leading to synapsis, novel DNA synthesis, strand dissolution, and repair. Many more proteins are involved in both NHEJ and HR, which are not depicted here for clarity, as they are not referred to in the main text. For details, see recent reviews by Lieber [81] and San Filippo *et al. *[80].

DNA DSBs can be induced by ionizing radiation and chemicals, or can occur as a consequence of replication failure. They represent a major challenge to cells, as they can directly lead to chromosome instability and large genetic alterations. DSBs are mainly repaired by either HR [80] or NHEJ [81] (Figure 3). HR is an error-free repair pathway that preferentially utilizes a sister chromatid as template, which is only present in late S- or G2-phase of the cell cycle, to repair DNA damage. By contrast, NHEJ occurs in all phases of the cell cycle, and fuses broken DNA ends together. Prior to NHEJ-associated ligation, limited DNA end-processing may occur, and this is usually associated with loss of a few nucleotides. NHEJ is therefore a more error-prone repair process than HR. Both DSB repair mechanisms begin with detection of the break and subsequent DNA processing, especially during HR, and these steps are associated with extensive phosphorylation and ubiquitylation events that modify the chromatin and proteins surrounding the break [15]. In the direct vicinity of the break, histone H2AX is phosphorylated by phosphatidylinositol 3-kinases such as ATM and ATR, leading to recruitment of mediator factors, including the scaffold protein MDC1 and the signaling proteins BRCA1 and 53BP1/TP53BP1 [4,82].

Chromatin structure plays a major role in DSB repair and signaling. The compaction of chromatin influences the sensitivity of cells to DSBs and the efficiency of the DDR [83]. Furthermore, breaks in heterochromatin are repaired more slowly than in euchromatin and require ATM-dependent phosphorylation of the heterochromatin protein KAP1/TRIM28 [84,85]. Moreover, it has been shown that chromatin becomes more relaxed upon DSB induction [85-87] and expands locally in an ATP-dependent fashion [88]. It thus appears that the effectiveness of the DSB-associated DDR is tightly regulated by the chromatin structure, and that the process itself modulates chromatin.

## ATP-dependent chromatin remodeling during DSB repair

One of the key proteins in HR, RAD54, contains a functional SWI2/SNF2 ATPase domain that is essential for several of the protein's functions [89,90]. For instance, through this domain RAD54 promotes branch migration of the holiday junction, an HR intermediate crossstranded DNA structure [90,91]. Additionally, many other SWI2/SNF2 domain-containing protein complexes function during the DSB-induced DDR (Table 1).

### SWI/SNF

The yeast SWI/SNF complex facilitates the pairing of homologous DNA strands during HR, and is recruited to sites of damage [92,93]. In addition, the SWI/SNF-related RSC complex in yeast is involved in DSB repair [92,94,95], particularly in HR, in which it is implicated in new DNA synthesis [92] and recombination between sister chromatids during S/G2 phase[ 96]. In addition, RSC functions in NHEJ, because recruitment of the core ATPase of RSC, Sth1, to DSBs depends on the NHEJ-specific DNA end-binding protein yKu70, and two of the RSC subunits interact with yKu80 [95,97]. Conversely, the recruitment of yKu70, the endo/exonuclease Mre11 and the damage-sensor kinases Mec1 and Tel1, and the phosphorylation of H2A at DSBs are also affected by RSC subunits [97,98]. During DSB repair, RSC locally increases DNA accessibility by nucleosome displacement at the site of damage [99], which may be a means through which RSC promotes multiple steps of NHEJ and HR.

**Figure 4:**
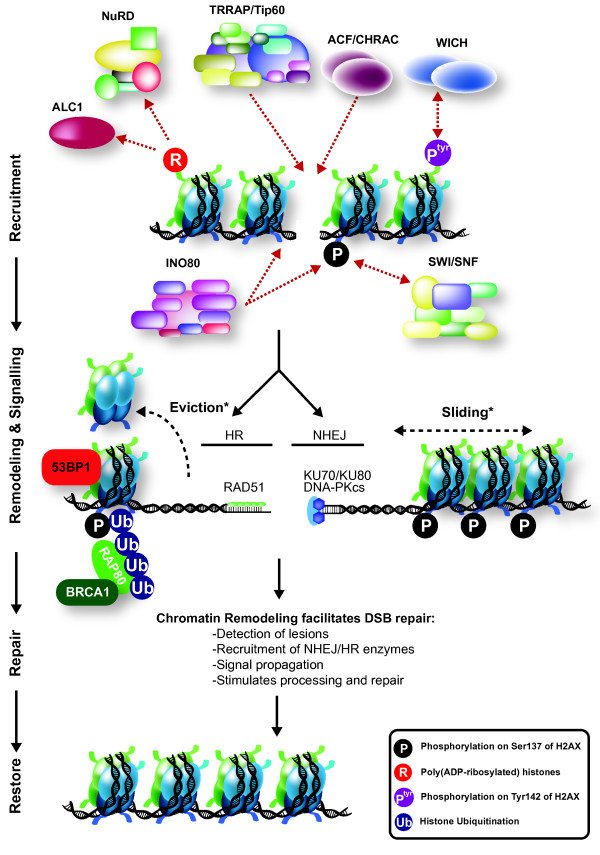
**ATP-dependent chromatin remodeling during mammalian double-strand break (DSB) repair**. A variety of ATP-dependent chromatin-remodeling complexes slide, evict, and modify nucleosomes to assist DSB repair, by increasing accessibility to DNA and facilitating DNA end-resection. Recruitment of various NHEJ, HR and signaling factors, and induction and maintenance of histone modifications at the site of damage depend on the recruitment and activity of various remodeling complexes. ALC1, which regulates DSB repair, and the NuRD complex, which promotes DSB repair and aids checkpoint activation, are both recruited in a poly(ADP ribose) polymerase (PARP)-dependent manner, which may involve poly(ADP-ribos)ylation of histones. The TRRAP/Tip60 complex localizes to DSBs to acetylate histones, and promote subsequent repair and signaling events. The ACF and/or CHRAC complexes are recruited to sites of damage, and interact with KU70 to facilitate efficient NHEJ. The WICH complex phosphorylates histone H2AX in a non--canonical manner, and stimulates efficient damage signaling and recruitment of repair factors. Finally, SWI/SNF interacts with acetylated histones H3 and H4 at the site of damage and, together with INO80, facilitates phosphorylation of histone H2AX. Red dotted arrows depict chromatin recruitment and protein-protein interactions. See main text for more detail and references. *Sliding and eviction mechanisms are probably involved in both NHEJ and HR.

Similarly, in mammals SWI/SNF seems to regulate DDR efficiency. Interference with the two SWI/SNF catalytic ATPase subunits BRG1 and BRM sensitizes cells to DSB-inducing agents, impairs damage-induced phosphorylation of H2AX, and decreases repair [100,101]. Both proteins are recruited to damaged chromatin, and interact with acetylated H3 and H4 in nucleosomes [102,103]. This interaction is stimulated by phosphorylation of H2AX, and depends on the DNA damage-induced activity of acetyltransferases Gcn5, CBP/CREBBP, and p300/EP300. SWI/SNF recruitment is also facilitated by an increased DNA damage-induced interaction with the early DDR protein BRIT1/MCPH1, which depends on phosphorylation of the SWI/SNF core protein BAF170 by ATM/ATR [104]. Furthermore, BRM is required for Ku70 recruitment to damaged DNA and thus for efficient NHEJ [103]. Together, these results suggest that mammalian SWI/SNF functions to facilitate DSB repair, and is recruited by and stimulates phosphorylation of H2AX (Figure 4).

### INO80

Multiple remodeling activities of INO80 family complexes have been reported to regulate different steps of the DSB-induced DDR. In yeast, the INO80 complex is recruited to DSBs in a H2A-phosphorylation-dependent manner and is required for removal of nucleosomes at DSB sites, affecting both HR and NHEJ [65,105-111]. Likewise, the INO80-related SWR1 complex is also recruited to DSBs in a H2A-phosphorylation-dependent manner, where it may function in nucleosome remodeling to regulate DSB repair [109,112,113]. INO80 and SWR1 probably function antagonistically in regulating genome integrity and checkpoint adaptation, which is the escape from an extended cell cycle checkpoint [112,114]. INO80 promotes genome stability and checkpoint escape by exchanging the SWR1-incorporated histone variant Htz for H2A. Other than in yeast, the mammalian INO80 complex is recruited to DSBs independently of phosphorylated H2AX and is implicated in HR [66,115], whereas another study has suggested that the core ATPase INO80 and the Arp5 subunit regulate phosphorylation of H2AX [116]. Furthermore, TIP49/RUVBL1 and TIP48/RUVBL2, core subunits of the different mammalian INO80 family complexes, affect RAD51 loading onto DSB sites during HR, but do not affect phosphorylation of H2AX [117]. These findings suggests that INO80 functions during different steps of HR. Apart from a direct role in DSB repair, INO80 might also indirectly function to regulate transcription of the RAD54 and XRCC3 DSB repair genes [118].

Several subunits of the mammalian SWR1-related TRRAP-Tip60 complex, including the ATPase p400/EP400 and the acetyltransferase Tip60/KAT5, are recruited to DSBs and locally decrease nucleosome stability [119-121]. Tip60 acetylates ATM and the histones H2A/H2AX and H4 after DNA damage, and is required for efficient DSB repair [120,122-125]. The activity of both p400 and Tip60 is required for subsequent steps of DSB repair, such as ubiquitylation of histones, and recruitment of the signaling proteins BRCA1 and 53BP1 [119,120]. Furthermore, in *Drosophila*, p400 and Tip60 were both shown to catalyze the exchange of the phosphorylated H2AX homolog H2Av for unmodified H2Av at sites of damage [125]. Together, these results indicate that different chromatin-remodeling complexes in the INO80 family perform different functions in DSB repair to make chromatin more accessible for repair and signaling factors via mechanisms of histone acetylation and nucleosome remodeling (Figure 4).

### CHD

Different members of the CHD family of chromatin remodelers are involved in regulating DSB repair in chromatin. It has been shown that CHD3 is dispersed from DSBs in heterochromatin via a mechanism involving KAP-1 phosphorylation by ATM, leading to chromatin relaxation and heterochromatic DSB repair [84]. CHD3 and the related CHD4 protein can both act as the central ATPase enzyme of the NuRD complex. Loss of several subunits of the CHD4-containing NuRD complex, including CHD4 itself, leads to increased ionizing radiation sensitivity in both *C. elegans *and mammalian cells and to defective DSB repair [126-130]. Furthermore, many subunits of NuRD are recruited to DSBs in a, at least for CHD4 and MTA1, poly(ADP ribose) polymerase (PARP)-dependent manner, but surprisingly, do not require phosphorylation of H2AX [128-131]. Additionally, CHD4 and the regulatory subunit MTA2 function in ubiquitin-mediated signaling by aiding the recruitment of the E3 ligases RNF168 and BRCA1 to DSBs [128,130]. Finally, CHD4 depletion leads to defects in the G1 to S transition, probably by controlling deacetylation of p53, and in the G2/M checkpoint upon DNA damage [128-130]. Together, these results suggest an important role for CHD4-containing NuRD in chromatin remodeling at the site of damage, facilitating efficient checkpoint activation (Figure 4).

Other members of the CHD family that are implicated in the DDR include the CHD1-like ATP-dependent chromatin-remodeling protein ALC1/CHD1L. ALC1 localizes to DSBs in a PARP-dependent manner, and binds the repair proteins Ku70, XRCC1 and DNA-PK [132,133]. ALC1 also interacts with the damage-recruited histone chaperone and DNA strand-break repair protein APLF [134], possibly to regulate NHEJ [135,136]. Finally, mouse cells deficient in CHD2 exhibit increased levels of spontaneous DNA damage, defective DNA-damage signaling, and sensitivity to DNA-damaging agents, suggesting a role for this protein in the DDR [137].

### ISWI

Multiple ISWI family chromatin-remodeling complexes function in different steps of HR, NHEJ and damage-associated signaling. Both of the ATPases of the ISWI family complexes, SNF2H and SNF2L/SMARCA1, are recruited to DSBs and confer sensitivity to DNA damage upon knockdown [71,72,126,138-141]. SNF2H is recruited to sites of damage via RNF20-mediated monoubiquitylation of H2B and methylation of H3K4 [141]. Depletion of SNF2H impairs DNA end-processing, RAD51 and BRCA1 recruitment, and subsequent repair via HR. Furthermore, the ACF1 protein, which forms part of the mammalian ACF and CHRAC complexes together with SNF2H, accumulates at DSBs and interacts with Ku70 [71,72]. Depletion of ACF1 and other members of the CHRAC complex leads to increased sensitivity to ionizing radiation, to defects in Ku70 and Ku80 recruitment and NHEJ, and to HR [72]. ACF1 is also implicated in controlling the G2/M checkpoint [71]. Another binding partner of SNF2H, WSTF/BAZ1B, which is part of the WICH complex, interacts with and phosporylates H2AX on Tyr142 instead of the canonical damage-induced phosphorylation of Ser139 [142]. Importantly, however, depletion of WSTF affects maintenance of H2AX-Ser139 phosporylation and recruitment of active ATM and MDC1 to DSBs. Like SNF2H and ACF1, WSTF is also recruited to DBSs [71]. Together, these data suggest that multiple ISWI chromatin-remodeling complexes act during DSB repair, probably to allow efficient recruitment and maintenance of repair proteins and signaling events (Figure 4).

### Discussion and perspectives

It is clear from the above overview that many different ATP-dependent chromatin-remodeling complexes participate to allow efficient DSB repair and signaling (Figure 4). Although some complexes have been implicated in specific repair events or found to be required for altering nucleosome occupancy of DNA surrounding DBSs, the specific activity of most complexes at sites of damage still remains unclear. Furthermore, contradictory results still cause an ongoing debate as to the exact contribution of these complexes to repair [68]. To resolve these issues, and to answer the emerging question why so many complexes are involved, future studies should be aimed at deciphering their specific molecular activity during subsequent repair steps, and at determining the interdependency of different remodeling processes. This will require a multidisciplinary combination of biochemical and cell-biological analysis, incorporating newly developed quantitative proteomic and microscopy techniques. Together, these studies should provide an integrated view on the function and interplay of the multiple chromatin-remodeling complexes and other chromatin modifications that aid repair efficiency.

## Conclusion

NER and DSB repair are in principle independent pathways, and are initiated by entirely distinct DNA damage-sensing mechanisms. Despite this difference, remarkable similar chromatin modifications (such as H2AX phosphorylation and histone ubiquitylation) and subsequent downstream signaling cascades are triggered by both processes [143-146]. It is surprising to find that, a similar set of upstream chromatin remodelers is used for both efficient repair of single-strand helix-distorting lesions by NER and for repair of DNA DSBs. This suggests that both for initiation and downstream signaling in distinct repair pathways, similar strategies are used. Despite these similarities, significant differences with respect to chromatin modification are also apparent between HR, NHEJ and NER, which are readily visible at the microscopic level [6]. Specifically, HR factors and downstream signaling molecules form large molecular assemblies on chromatin, referred to as ionizing radiation induced foci, which include chromatin changes that are many kilobases to megabases in length [147,148]. These microscopic foci are thought to be assembled around DSBs. Strikingly, neither NHEJ nor NER factors form such foci around lesions [6,15,149]. Thus, it seems that more or higher-order chromatin modifications are required to allow the complex HR-associated DNA rearrangements, such as homology search, strand annealing, branch migration, and resolution, to occur. This may also be the reason why there are more diverse remodeling complexes in DSB repair. In addition, the repair-patch sizes of NER events are typically around 25 to 30 nucleotides, which is within the range of a single nucleosome, whereas HR involves DNA end-resection and resynthesis of hundreds to thousands of base pairs; therefore it is likely that the latter repair pathway requires more robust chromatin remodeling.

One of the challenges ahead will be to determine at which steps all the different chromatin remodelers actually function in the DDR (either upstream or downstream of repair and/or signaling), and to what extent they function redundantly or influence each other's function. To obtain an integral view on chromatin remodeling associated with the DDR, a systems-biology approach should be applied, by combining detailed genetic and phenotypic analysis with genomic procedures. Because most of the chromatin remodelers have additional functions outside DDR, particularly in transcription regulation, both genome-wide position and gene-expression analysis will be confounded by the fact that an important part of the DDR network is the transcriptional response branch. Although both transcription and DDR require ATP-dependent chromatin remodeling, both chromatin-associated processes are conceptually rather different. Transcription activation occurs in a site-specific and time-dependent manner, and occurs on a gene with an inherent chromatin structure or compaction. By contrast, repair must be able to occur on all genomic locations, at any time [150]. In transcription regulation, the sequential hierarchy by which chromatin structure is altered is difficult to define. As repair takes place at rather diverse chromatin structures, the general concepts and hierarchical molecular mechanism may perhaps be even more difficult to define.

It is likely that many chromatin-remodeling complexes have thus far escaped scrutiny because of redundancy or because they only function in specific chromatin environments, which may not lead to a phenotype that is readily identifiable by genetic screens. Future research should aim to determine whether different complexes are involved in repair of different types of chromatin or in different phases of the cell cycle. In addition, as the DDR is tightly regulated in different cell types involving alternative repair pathways, DDR-associated chromatin changes may also be cell-type-specific [20,151]. It will be interesting to determine to what extent the chromatin landscape and, more specifically, ATP-dependent chromatin-remodeling factors, contribute to DDR differences of different cell types. For instance, we have recently shown that in *C. elegans*, some SWI/SNF chromatin-remodeling subunits are required only for the response of germ cells to UV, while others play a role only in somatic cells (unpublished results; [64]). Some of the contradictory results or interspecies differences observed to date may in fact reflect such differences in cell type and chromatin involvement. Eventually, knowledge about the role that chromatin plays in the DDR might also help us to better understand the etiology of certain cancers. Both ATP-dependent chromatin remodeling [152-155] and the DDR [3,5] are implicated in tumorigenesis, but their interplay in this process is still not understood.

## List of abbreviations

64PP: 6-4 photoproducts; CPD: cyclobutane pyrimidine dimer; DDR: DNA-damage response; DSB: double-strand break; GG-NER: global genome nucleotide excision repair; HR: homologous repair; NER: nucleotide excision repair; NHEJ: non--homologous end-joining; PARP: poly(ADP ribose) polymerase; TC-NER: transcription-coupled nucleotide excision repair.

## Competing interests

The authors declare that they have no competing interests.

## Authors' contributions

HL drafted and edited the manuscript. JAM edited the manuscript and provided artwork. WV helped drafting and edited the manuscript. All authors have read and approved the final manuscript.
